# The acetabular labrum—rising from obscurity

**DOI:** 10.1093/jhps/hnag002

**Published:** 2026-02-10

**Authors:** Richard E Field

**Affiliations:** City St George’s University of London, London, United Kingdom

As a child, I would sometimes sneak into my parents’ library to look at the pictures in my mother’s 1949, 30th edition of Gray’s anatomy [1]. At the age of six, midline structures most appealed to my curiosity, and it was almost two decades before my attention shifted laterally to the nearby ball-and-socket joint. As a newly qualified doctor studying for my Primary Fellowship of the Royal College of Surgeons, I spent long hours trying to understand and memorize the contents of the 6th Edition of Last’s Anatomy [2]. Near the end of the second column of page 149, Last describes the acetabulum and devotes two short sentences to the ‘labrum acetabulare’. He observes that the labrum comprises ‘… *a rim of dense fibrous tissue which encloses the femoral head beyond its equator, thus increasing the stability of the joint*’. As a trainee Orthopaedic surgeon, I acquired a second-hand copy of the 6th Edition of Campbell’s Operative Orthopaedics [3]. Chapter 9 is devoted the management of traumatic affections of joints. In this chapter’s 157-pages, numerous shoulder, knee, and ankle interventions are described. Only one page [3] is assigned to the hip, with two fleeting references to labral pathology. The first is Attenberg’s description of three patients who developed severe pain and arthrosis secondary to a torn labrum, all of whom enjoyed a good result from the excision of the loose fragment [4]. The second is a personal communication from Ingram [5], who had identified a detached labrum and capsule as the cause of a case of recurrent dislocation of the hip and had reported that reattachment of these structures to the acetabular rim had proven curative.

At the start of my career, it would have been fanciful to suggest that before my retirement, mighty tomes [6–8] would be published on hip preservation surgery and that hundreds of surgeons would devote their careers to this new subspeciality. Equally absurd would have been the suggestion that tens of thousands of patients would present to our clinics with MRI reports detailing a torn labrum and an expectation that surgical repair should be undertaken.

One of the challenges facing clinicians is to convince patients that the isolated repair of a torn labrum will not address their underlying problem, and that the cause of their labral trauma needs to be addressed to achieve a good clinical outcome. Likewise, clinicians need to identify when a patient has passed beyond the point where hip preservation surgery can successfully address their problem. Also, patients need to be aware that, with advancing age, there is an increasing likelihood of associated degenerative changes that may preclude successful joint preservation.

In 2015, Domb and his colleagues reported that when patients with a Tönnis grade of 0 or 1 were treated using an arthroscopic intervention, within 2 years of surgery, those who were over the age of 50 had a higher risk of progression to hip replacement (17.3%) than patients under the age of 30 (1.9%). However, on a more positive note, the older patients who did not progress to hip replacement enjoyed similar benefits from the arthroscopic procedures as the younger patients [9].

In 2020, White and his colleagues in Englewood, Colorado, reported that for patients over 40, labral repair was three times more likely to fail than labral reconstruction [10]. In this issue, we present further work from White and his team [11] that looks at the outcome of labral reconstruction in the management of patients over the age of 60, with Tönnis grade 0 or 1 changes. Once again, the results are encouraging, and it will be interesting to see whether the strategy of reconstituting the labral seal, with the sacrifice of the highly innovated native labrum, will gain greater traction among the hip preservation community.

In contrast to White’s strategy of labral reconstruction, we also present a study from Nikou and his colleagues in Gothenburg, Sweden [12], reporting their results in treating patients with labral ossification. In Nikou’s study, the patients were relatively mature, with an average age of 47.7 years, and a standard deviation of 8.1 years. Unlike White’s patients, the labrum was either debrided or repaired. In Nikou’s group, 25% of the cases progressed to total hip replacement within 2 years. However, five of these six cases started with Tönnis grade 2 or 3 changes; so, it remains unknown whether restoration of the suction seal might have altered the outcome or whether the progression to hip replacement was driven by pre-existing, irreversible degenerative changes.

If the efficacy of labral reconstruction is accepted and adopted by the hip preservation community, the tissue used to reconstruct the labrum will become a topic of interest. The study from Richards and the team in Columbia, Missouri [13], provides a valuable step to better understand the biology of labral tissue and a methodology to help researchers identify the potential strengths and weaknesses of different tissue options. Richards’ study compares meniscal tissue, acetabular labral tissue, and anterior tibialis tendon. Hopefully, future work will provide similar insights into the biology of the fascia lata used by White and other allograft alternatives in current use. Perhaps it is not fanciful to foresee a time when synthetic labral grafts will be stocked on the implant shelf of our operating theatres.

We hope that you find these studies and the other studies presented in issue 13.1 informative, and that they stimulate you to develop your practice through the coming years.
